# Supplementary material for: CDK12 is hyperactivated and a synthetic-lethal target in BRAF-mutated melanoma
Source: Nat Commun. 2022 Oct 29;13:6457. doi: 10.1038/s41467-022-34179-8 (PMC9617877; doi:10.1038/s41467-022-34179-8)
Supplement: Supplementary file 1 — Supplementary Information [file 41467_2022_34179_MOESM1_ESM.pdf]

Supplementary Information

CDK12 is hyperactivated and a synthetic-lethal target in BRAF-mutated melanoma

Thibault Houles, Geneviève Lavoie, Sami Nourreddine, Winnie Cheung, Éric Vaillancourt-Jean, Célia M. Guérin, Mathieu Bouttier, Benoit Grondin, Sichun Lin, Marc K. Saba-El-Leil, Stephane Angers, Sylvain Meloche, Philippe P. Roux

SUPPLEMENTARY FIGURES

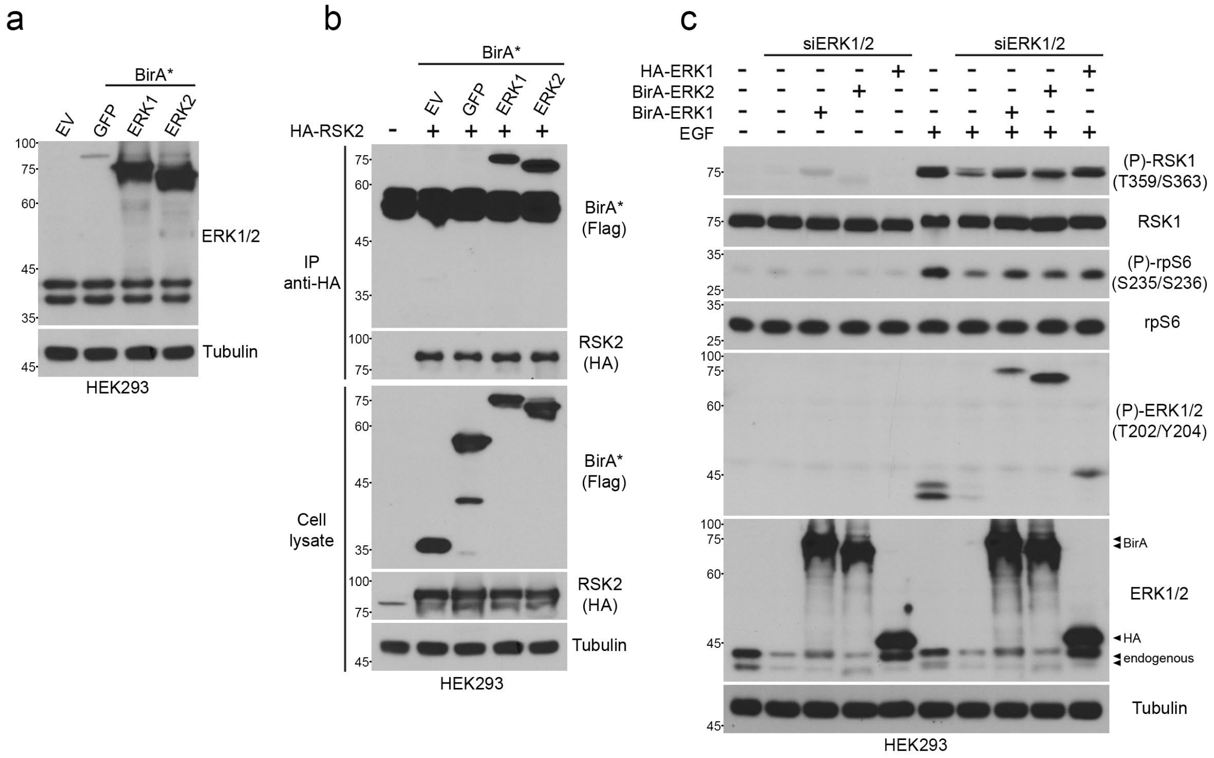

Supplementary Figure 1: Validation of BirA-ERK1/2 functionality.

a The expression of each transfected bait (BirA-GFP, BirA-ERK1 and BirA-ERK2) and endogenous ERK1/2 was assessed by immunoblotting in HEK293 cell lysates. **b** Immunoprecipitation of each transfected bait (BirA, BirA-GFP, BirA-ERK1 and BirA-ERK2) with HA-RSK2. HEK293 cells were transfected with different expression constructs and immunoprecipitated with HA antibodies. Co-immunoprecipitation of BirA-fusion proteins was assessed by immunoblotting with an antibody against the Flag epitope. **c** Immunoblot depicting the activity of each transfected bait (BirA-GFP, BirA-ERK1, BirA-ERK2 and HA-ERK1) against RSK1, an established ERK1/2 substrate. HEK293 cells were co-transfected with each bait and ERK1/2 siRNAs (siERK1/2), serum-starved overnight, and stimulated for 10 min with EGF (25

ng/ml). Representative data of 3 independent experiments (n = 3). Source data are provided as a Source Data file.

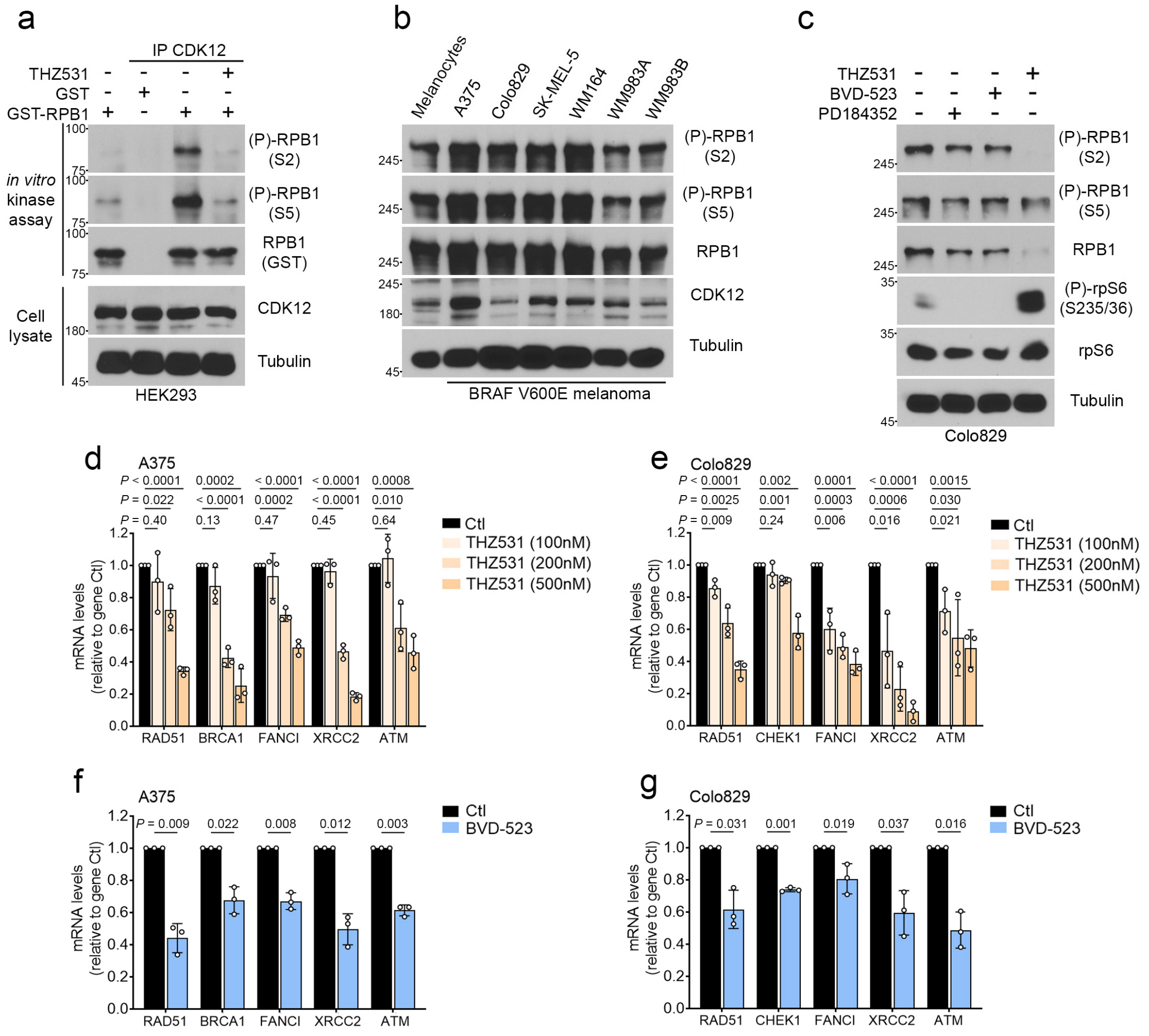

Supplementary Figure 2: Correlation between CDK12 and ERK1/2 in BRAF-mutated melanoma cells.

a *In vitro* kinase assay based on endogenous CDK12 immunoprecipitated from HEK293 and incubated with recombinant GST or GST-RPB1 and THZ531 (500 nM). Samples were immunoblotted with specific RPB1 phospho-Ser2/Ser5 and GST antibodies. **b** Immunoblot depicting CDK12, as well as total and phosphorylated RPB1, in serum-starved melanocytes and indicated melanoma cell lines. **c** Immunoblot of Colo829 cells serum-starved overnight, and treated with BVD-523 (5 µM), PD184352 (10 µM) or THZ531 (500 nM) for 6 h**.** **d**, **e** qPCR of A375

(**d**) or Colo829 (**e**) cells treated with THZ531 (100, 200 and 500 nM) for 6 h. **f**, **g** qPCR of A375 (**f**) or Colo829 (**g**) cells treated with BVD-523 (5 µM) for 6 h. Data are represented as mean ± SD of 3 independent experiments (n = 3) (**d**-**g**). (**a**-**c**) Representative data of 3 independent experiments (n = 3). Significance was determined using unpaired two-tailed Student's t-tests. Source data are provided as a Source Data file.

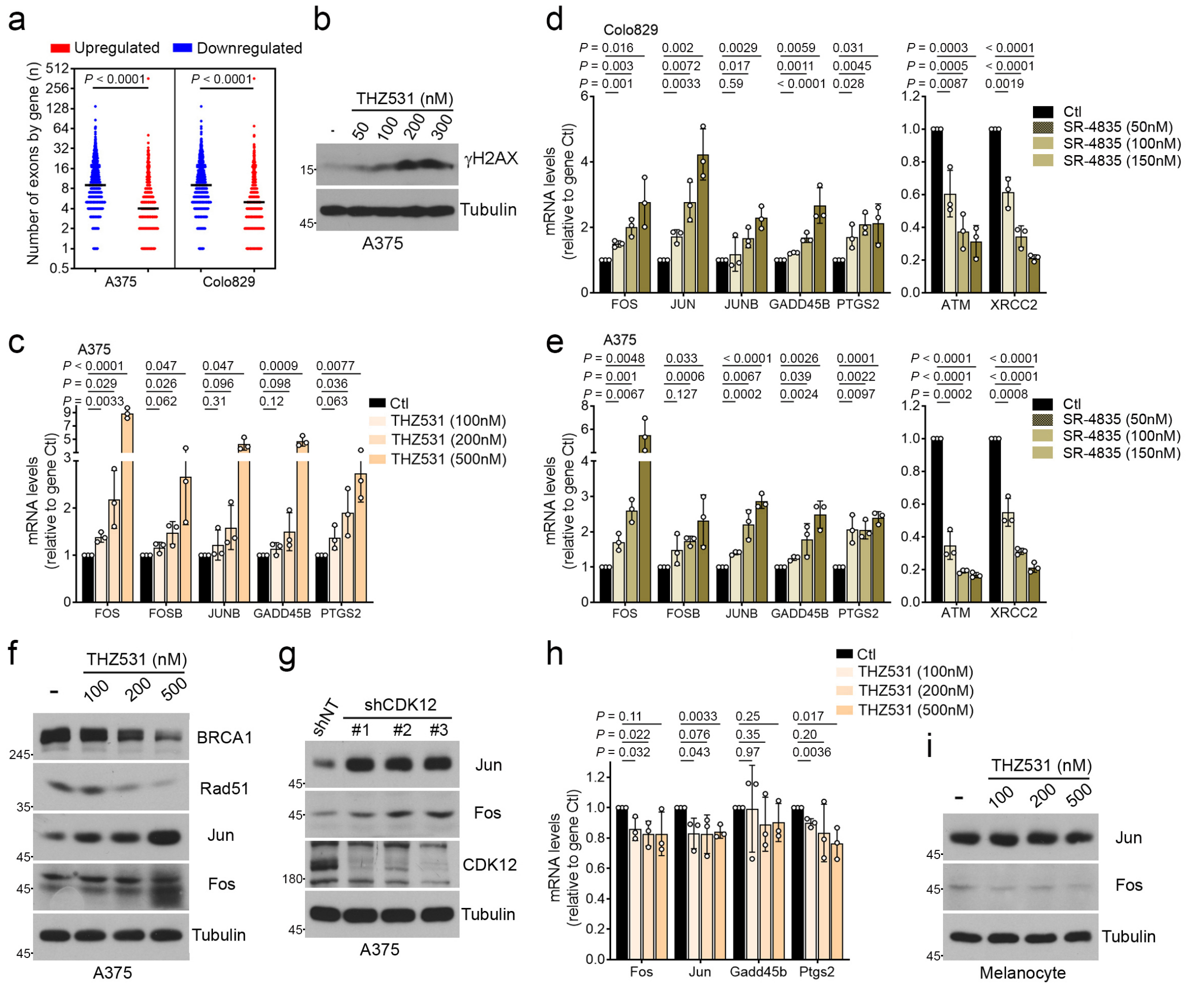

Supplementary Figure 3: CDK12 regulates genes according to their size and number of exons in BRAF-mutated melanoma cells.

a Number of exons of significantly upregulated and downregulated genes in A375 and Colo829 cells. **b** Immunoblot depicting γH2AX levels in A375 cells treated for 24 h with increasing concentrations of THZ531 (0, 50, 100, 200 and 300 nM). **c** qPCR of A375 cells treated for 6 h with THZ531 (100, 200, 500 nM). **d, e** qPCR of Colo829 (**d**) or A375 (**e**) cells treated for 6 h with increasing concentrations of SR-4835 (50, 100, 150 nM). **f** Immunoblot of A375 cells treated for 12 h with THZ531 (100, 200, 500 nM). **g** Immunoblot of A375 cells infected with lentiviral CDK12 shRNAs (shCDK12) for 72 h. **h** qPCR of melanocytes treated for 6 h with THZ531 (100, 200, 500

nM). **i** Immunoblot of melanocytes treated for 12 h with THZ531 (100, 200, 500 nM). Data are represented as mean ± SD of independent experiments, n = 3 (**c-e, h**) or number of exons per deregulated gene from n = 3 independent biological replicates for each cell line (**a**). (**b, f, g, i**) Representative data of 3 independent experiments (n = 3). Significance was determined using unpaired two-tailed Student's t-tests (**a, c-e, h**). Source data are provided as a Source Data file.

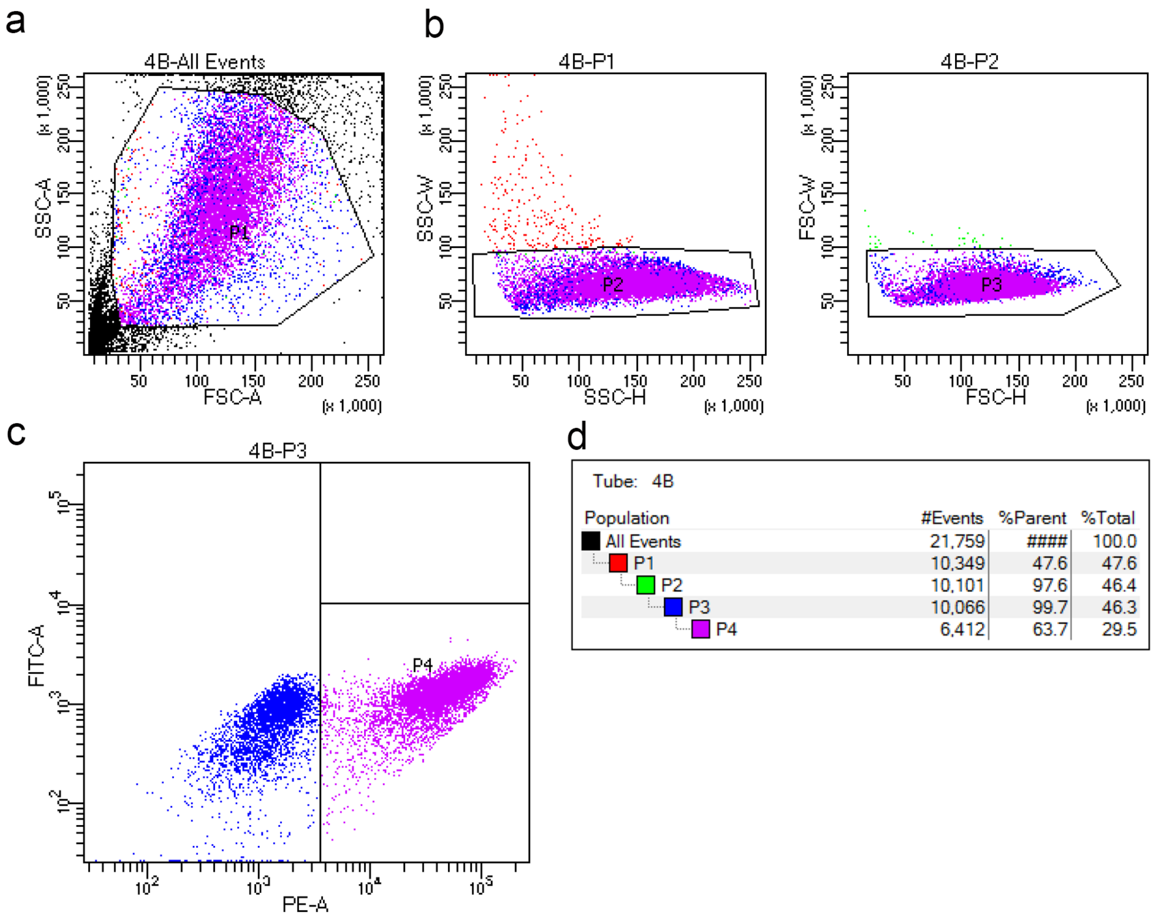

Tube: 4B

Population	#Events	%Parent	%Total
All Events	21,759	####	100.0
P1	10,349	47.6	47.6
P2	10,101	97.6	46.4
P3	10,066	99.7	46.3
P4	6,412	63.7	29.5

Supplementary Figure 4: Gating strategy for flow cytometry.

Representation of the gating strategy used for Figs. 5 and 6. **a** P1 is first selected from FSC-A versus SSC-A plot. **b** Doublets are removed from P1 population after gating for P2 from SSC-W versus SSC-H plot, and P3 from FSC-W versus FSC-H plot. **c** From P3 a dot plot is generated by plotting FITC-A versus PE-A, ANNEXIN-V+ cells are determined by gating for P4. **d** representative calculation using gating strategy.
