# Supplementary material for: CDK12 is hyperactivated and a synthetic-lethal target in BRAF-mutated melanoma
Source: Nat Commun. 2022 Oct 29;13:6457. doi: 10.1038/s41467-022-34179-8 (PMC9617877; doi:10.1038/s41467-022-34179-8)
Supplement: Supplementary file 2 — Description of Additional Supplementary Files [file 41467_2022_34179_MOESM2_ESM.pdf]

File Name: **Supplementary Data 1**

Description: **BioID dataset from HEK293 cell lines expressing BirA*-ERK1 or ERK2.**

File Name: **Supplementary Data 2**

Description: **Transcriptome of A375 and Colo829 cells treated with THZ531 (500 nM) for 6 hours compared to untreated cells.**
